# Using Confidence Interval-Based Estimation of Relevance to Select Social-Cognitive Determinants for Behavior Change Interventions

**DOI:** 10.3389/fpubh.2017.00165

**Published:** 2017-07-13

**Authors:** Rik Crutzen, Gjalt-Jorn Ygram Peters, Judith Noijen

**Affiliations:** ^1^Department of Health Promotion, Maastricht University/CAPHRI, Maastricht, Netherlands; ^2^Faculty of Psychology and Education Science, Open University of the Netherlands, Heerlen, Netherlands; ^3^Faculty of Psychology and Neuroscience, Department of Work and Social Psychology, Maastricht University, Maastricht, Netherlands; ^4^Jellinek Preventie, Arkin, Amsterdam, Netherlands

**Keywords:** determinants, beliefs, behavior change, methods, intervention development, Confidence Interval-Based Estimation of Relevance

## Abstract

When developing an intervention aimed at behavior change, one of the crucial steps in the development process is to select the most relevant social-cognitive determinants. These determinants can be seen as the buttons one needs to push to establish behavior change. Insight into these determinants is needed to select behavior change methods (i.e., general behavior change techniques that are applied in an intervention) in the development process. Therefore, a study on determinants is often conducted as formative research in the intervention development process. Ideally, all relevant determinants identified in such a study are addressed by an intervention. However, when developing a behavior change intervention, there are limits in terms of, for example, resources available for intervention development and the amount of content that participants of an intervention can be exposed to. Hence, it is important to select those determinants that are most relevant to the target behavior as these determinants should be addressed in an intervention. The aim of the current paper is to introduce a novel approach to select the most relevant social-cognitive determinants and use them in intervention development. This approach is based on visualization of confidence intervals for the means and correlation coefficients for all determinants simultaneously. This visualization facilitates comparison, which is necessary when making selections. By means of a case study on the determinants of using a high dose of 3,4-methylenedioxymethamphetamine (commonly known as ecstasy), we illustrate this approach. We provide a freely available tool to facilitate the analyses needed in this approach.

## Introduction

When developing an intervention aimed at behavior change, one of the crucial steps in the development process is to select the most relevant determinants (1). In lay terms, these determinants are the closest approximation to “the buttons one needs to push” to establish behavior change. Insight into these determinants is needed to select behavior change methods (i.e., general behavior change techniques that are applied in an intervention) in the development process. The aim of the current paper is to introduce a novel approach to select the most relevant determinants and use them in intervention development.

There are three main types of variables that have an influence on behavior: environmental, genetic, and psychological variables. When developing an intervention aimed at behavior change, the focus is mostly on psychological variables. First, because these variables are most likely to be changeable by an intervention (2). Second, because overt behavior results from neural activation patterns (3). Hence, all overt behavior is necessarily caused by psychological variables in all conceivable cases except physical coercion. In other words: all environmental (e.g., social or physical) and genetic influences on behavior eventually operate through (and manifest as) psychological variables (4),^1^ of which, in the context of behavior change, social-cognitive determinants have received the most attention.

Theories aiming to explain behavior, such as the Reasoned Action Approach [RAA (5)], the Health Belief Model [HBM (6)], and the Extended Parallel Process Model [EPPM (7)], postulate specific social-cognitive determinants and their relationships to each other and behavior. Each of these theories applies to specific (antecedents of) behaviors: for example, the RAA explains reasoned action, the HBM health behavior, and the EPPM the processing of threatening communication. This property of theories (i.e., dealing with bounded aspects of reality) is not a shortcoming, but in line with the definition of theories as reductions of reality, which is also emphasized by Occam’s razor (8). This means that to obtain the most exhaustive understanding of which psychological variables determine a behavior, it will often be necessary to combine several theories of behavior explanation (9).

### Identifying Determinants at Different Levels of Psychological Aggregation

In intervention planning, this combination of theories informs the so-called logic model of change (1). Such a logic model contains, for a specific behavior in a specific target population, what is known about the psychological variables and environmental conditions that predict the behavior. For each relevant environmental condition, environmental agents who control the condition are identified, and an intervention may then be developed for each of them (10). Because targeting those environmental conditions occurs through targeting the determinants of the relevant agents, the process of selecting relevant determinants for environmental agents is comparable to selecting relevant determinants for the target population (1). It is important to note that these determinants have a given level of psychological aggregation. For example, in the RAA, behavior is the highest level of psychological aggregation (level 1), followed by intention (level 2), attitude (level 3), experiential attitude (level 4) and behavioral beliefs (level 5) (5, 9). In other words, determinants of behavior can be organized on the aggregation level hierarchy in terms of specificity versus generality, and various sub-determinants can often be distinguished for any determinant. For example, behavioral beliefs are sub-determinants of attitude. In this paper, we use “sub-determinants” to refer to determinants at a lower level of psychological aggregation.

The overarching determinants at higher levels of generality (e.g., attitude and self-efficacy) are those needed to select appropriate behavior change methods. Behavior change methods have different components and are not equally effective for all determinants. For example, while stimulating enactive mastery experiences can be used to improve self-efficacy, it is less suitable to foster attitude change. We refer to Kok et al. (11) for an overview of behavior change methods linked to specific determinants.

Sub-determinants formulated at a high level of specificity (e.g., beliefs in the case of RAA) are crucial when studying determinants, because those very specific aspects are what is used in operationalizations and intervention messages. As operationalizations are stimuli that people process (and respond to, for example, items in a questionnaire), they need to have sufficient specificity to relate to real-world phenomena (e.g., a questionnaire item in ordinary language). Similarly, as intervention messages will necessarily address more or less tangible aspects of reality, these, too, are based on sufficiently specific sub-determinants. For example, even though the RAA postulates that intention is the most proximal determinant of behavior, intervention messages mostly concern beliefs underlying, for example, attitude (e.g., “being physical active is enjoyable”) or perceived norm (e.g., “X% of people your age adhere to recommended levels of physical activity”).

Thus, before developing a behavior change intervention, it is important to establish the determinants and underlying sub-determinants that predict the target behavior. This is an essential part of the needs assessment, which ultimately results in the logic model of change, in Intervention Mapping (1) as well as other frameworks (12). Therefore, a determinant study is often conducted as formative research in the intervention development process. When conducting such a study, it is important to include all possible sub-determinants that might be relevant for the target behavior of the intervention. Using the core processes (i.e., the processes involved in understanding a problem or answering a question with empirical data and theory) is critical to identify sub-determinants at all levels of aggregation (11). The first step in using the core processes is to conduct a brainstorm about possible sub-determinants for the specific behavior and the specific target group. The second step is to gather evidence from previous empirical studies. It is important to stress that different (but complementary) types of studies can be used. For example, while a meta-analysis can provide evidence for the strength of the association between higher level determinants (e.g., self-efficacy) and the target behavior, an interview study can provide more in-depth insight into lower level determinants (e.g., specific situations in which target population members exhibit low levels of self-efficacy). The third step is to use insights from psychological theories (7). The fourth step is to collect new empirical data that are specific to the target population, context, and behavior at hand. In this step, different types of studies can be conducted as well. For example, Peters (13) provides a practical guide regarding synthesizing previous literature and qualitative exploration of (sub-)determinants.

Subsequently, one needs to establish which of the potential (sub-)determinants are the most relevant given the target behavior, population, and context. This is important because practical considerations prohibit targeting all (sub-)determinants. For example, there are limits in terms of resources available for intervention development and the amount of content that participants of an intervention can be exposed to. To optimize intervention effectiveness, the selection of which (sub-)determinants will be targeted by an intervention should be guided by (sub-)determinant relevance. This paper focuses on establishing relevance based on data that are collected by means of surveys.

### Approaches to Establishing Relevance

Establishing relevance of determinants is a crucial step in the planning of behavior change interventions; however, as yet, no guidelines exist for establishing relevance of determinants. Due to a lack of clear guidelines, a variety of methods is used. For example, dichotomizing (a determinant of) behavior and then comparing means of (sub-)determinants; computing correlation coefficients for the association between (sub-)determinants and (a determinant of) behavior; or conducting regression analyses where (a determinant of) behavior is regressed on relevant (sub-)determinants [for some examples pertinent to the current subject matter, see, e.g., Ref. (14–18)]. These approaches combine two types of analyses: (1) assessing the univariate distribution of each (sub-)determinant and (2) assessing associations to behavior and/or determinants of behavior.

Assessing the associations of (sub-)determinants with behavior and/or determinants is important: those sub-determinants that are not associated to behavior and/or more proximal determinants will often be the least likely candidates to intervene upon. The univariate distributions are important because bimodal distributions may be indicative of subgroups, and strongly skewed distributions have implications for how a (sub-)determinant should be targeted. For example, if a sub-determinant is positively associated with behavior but left-skewed, most population members already have the desired value, so an intervention developer will want to reinforce it. Conversely, right-skewed positively associated sub-determinants imply a need for change, as most population members do not have the desired value yet. This latter category of sub-determinants would be more viable intervention targets, should a choice have to be made: there is more room for improvement.

Although these conventionally employed analyses have sensible aims, the analyses employed to achieve those aims are problematic. Regression analyses, for example, are useful to obtain a measure of the total explained variance in an outcome (e.g., *R*^2^) based on the sub-determinants included in a model. However, the regression coefficients provide little information as to determinant relevance because they are conditional upon the other predictors in the specific model (13, 19–21). These problems are resolved when looking at bivariate associations, but the common practice of dichotomizing behavior or a proximal determinant such as intention leads to information loss and underestimation of variation (22–24). Furthermore, Cohen’s *d* point estimates of differences between groups (e.g., intenders and non-intenders) can vary substantially from sample to sample (25), rendering them unfit for determinant selection on the basis of one sample. Although to a lesser extent, the same is true for estimates of means and correlation coefficients (26). Instead resorting to basing conclusions on *p*-values from null hypothesis significance tests is also widely discouraged (27–31). Using a frequentist approach, the most widely accepted method would be to base these decisions on the *confidence intervals* for the means and correlation coefficients.

However, such an approach is problematic because it requires intervention developers to parse a large amount of information simultaneously. For each (sub-)determinant, the univariate distribution and mean, as well the lower and upper confidence interval bounds would have to be inspected, as well as the correlation coefficients with behavior and perhaps a proximal determinant of behavior such as intention, again together with the lower and upper confidence interval bounds. Even with only 10 (sub-)determinants, and even if associations with a proximal determinant are not considered, this would mean researchers would have to simultaneously evaluate 60 estimates. The main challenge, therefore, is to find a method of assessing this large amount of information simultaneously. This is the challenge we aim to undertake with the presently proposed Confidence Interval-Based Estimation of Relevance (CIBER) approach.

### Confidence Interval-Based Estimation of Relevance

The presently proposed CIBER approach is based on data visualization. Visualizing the relevant data has three advantages. First, visualization enables mapping the data onto spatial dimensions, facilitating comparison, which is necessary when making selections. Second, visualization foregoes the seeming accuracy and objectivity afforded by numbers (32). Given the relative width of most sampling distributions and the subsequent variation that occurs in estimates over samples (25, 26), caution in basing decisions on the exact computed numbers seems prudent. Third, visualization enables assessing confidence intervals for means in the context of the raw data.

In the visual representations used in the CIBER approach, confidence intervals are represented using the diamond shapes commonly used for the aggregated effect size in meta-analyses (32). Unlike error bars with whiskers, diamonds do not draw attention to the confidence interval bounds. They are an efficient method of representing both the mean and the confidence interval in one shape, allowing both stroke and fill colors, which makes it possible to use the fill color to further facilitate interpretation, and the stroke color to identify, for example, which determinant a shape represents. Another advantage is that it is not easy to see the exact values of the three estimates represented by the diamond (the mean and lower an upper confidence bounds). Although this might not seem like an advantage at first glance, this lack of clarity is consistent with the estimates’ imprecision [i.e., their variation from sample to sample (32)]. These diamond plots are then used to visualize the raw data, the point estimate and confidence interval for the mean, and the point estimate and confidence interval for the correlation with behavior and/or one or several determinants of behavior. Each (sub-)determinant, the question used to assess it, as well as the anchors can be shown.

In other words, the CIBER approach acknowledges that several metrics need to be combined (correlation coefficients, means, and confidence intervals of both) and interpreted in order for data to become valuable information. In the next section, we will illustrate this approach by means of a case study on the determinants of using a high dose of 3,4-methylenedioxymethamphetamine (MDMA, commonly known as ecstasy).

## Case Study

In the Netherlands, MDMA content of ecstasy pills has gradually increased (33, 34), and the likely association of dose to risk (35) warrants intervention efforts to discourage using a high dose of MDMA. In our illustrations, we will use data collected in a determinant study designed to inform such intervention efforts. This study is conducted as part of the Party Panel initiative (for more information, see http://partypanel.eu/info). The full determinant study will be described elsewhere,^2^ but the most relevant information for the current paper is provided here. The materials and analysis script for this study have been made available at the Open Science Framework repository at https://osf.io/qf3sq. The data are under embargo until July 1, 2017, but will then be added to that repository. These efforts are taken to acknowledge a recent call for full disclosure to maximize scrutiny, foster accurate replication, and facilitate future data syntheses (e.g., meta-analyses) (36, 37).

### Recruitment, Procedure, Participants, and Ethical Approval

Participants were recruited through social media posts by Dutch nightlife prevention campaign Celebrate Safe and partner organizations and one funded Facebook post. Participation was voluntary, and no incentive was offered.

Participants visited http://partypanel.nl where they could open the survey. The survey was developed in LimeSurvey (38) and hosted on a secure TIER3+ server in the Netherlands. After providing online informed consent, participants completed the questions in the survey. These questions were mainly based on RAA (5). In addition, a number of target population members, specifically peers from the Amsterdam-based peer education project Unity, completed an online questionnaire with open-ended questions prompting for potential reasons for performing (or not performing) certain target behaviors (e.g., using a high dose of MDMA). This questionnaire was designed to approximate a belief elicitation procedure (5). The resulting beliefs were integrated in the questionnaire for this study (see Operationalizations).

The data presently analyzed were provided by 227 participants (all MDMA users, because only they could answer questions about their MDMA use). Of these, 203 reached the section where demographics were assessed, where 60% indicated they were male, 39% female, and 1% did not answer or indicated not identifying as male or female. The mean age was 25 years (SD = 7.0).

Ethical approval was provided by the ethical committee of the Dutch Open University (the form and the approval letter are available at https://osf.io/qf3sq).

### Operationalizations

**Figure 1:**
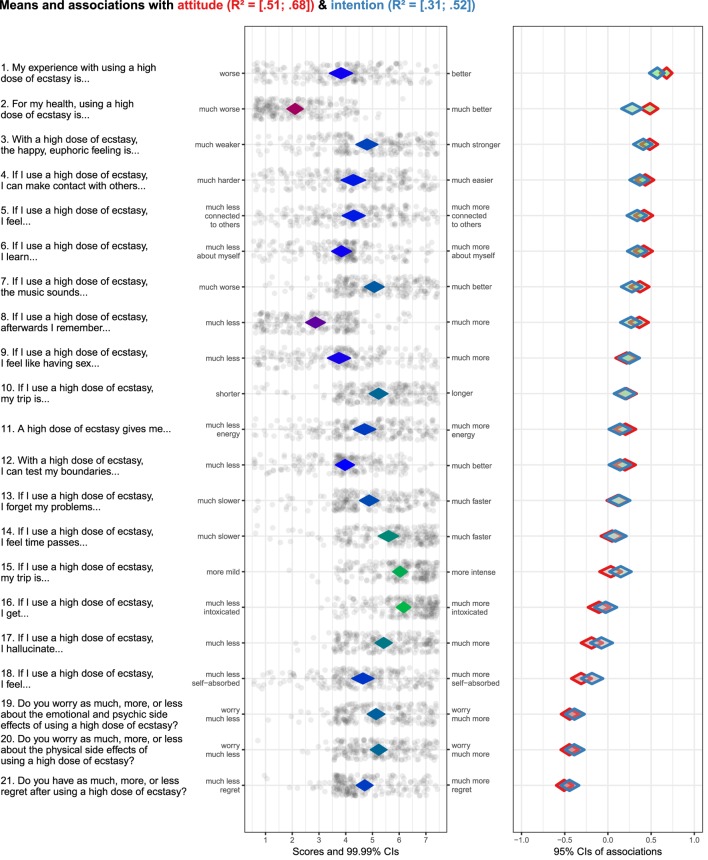
Output of case study regarding sub-determinants of attitude and intention to use a high dose of ecstasy. (Diamond fill color in left hand panel is indicative of items means: the redder the diamonds are, the lower the item means; the greener the diamonds are, the higher the item means. Diamond stroke color in right hand panel is used to differentiate between determinants. Diamond fill color in right hand panel is indicative of association strengths and their direction: the redder the diamonds are, the stronger and more negative the associations are; the greener the diamonds are, the stronger and more positive the associations are.).

All operationalizations (i.e., questions in the survey) were originally in Dutch. These original questions are available at https://osf.io/qf3sq and translations will be provided here. Please note that the term “ecstasy” is used in the translation of the questions to English and the remainder of this paper, as this more closely resembles the original questions in Dutch. In this case study, behavioral beliefs are associated with both attitude and intention. All questions regarding behavioral beliefs, attitude, and intention used 7-point response scales with varying anchors per question. Intention to use a high dose of ecstasy was measured with three questions assessing what in English could be described as participants’ intention, motivation, and expectation. The direct measure of attitude was a semantic differential where participants indicated what they thought about using highly dosed ecstasy pills if they used ecstasy, on the dimensions bad versus good, unpleasant versus pleasant, stupid versus smart, unhealthy versus healthy, and boring versus exciting. The behavioral beliefs, specifically the expectancies, were measured with a series of 21 items, each expressing a potential belief about using a high dose of ecstasy. The items and anchors can be seen in Figure 1.

### Output

Figure 1 provides the output following the proposed analytical approach. The items that were used to assess the behavioral beliefs (i.e., the sub-determinants in this case study) are shown to the left of the left hand panel. The anchors of the items are on the side of the left hand panel. The diamonds in the left hand panel show the item means with 99.99% confidence intervals. The fill color of the diamonds is indicative of the item means—the redder the diamonds are, the lower the item means; the greener the diamonds are, the higher the items means (blue denotes means in the middle of the scale). The dots surrounding the diamonds show the item scores of all participants with jitter added to prevent overplotting. The diamonds on the right hand panel show the association strengths (i.e., correlation coefficients with 95% confidence intervals) between individual items and determinants at different levels of psychological aggregation (attitude and intention in this example). The fill color of the diamonds is indicative of the association strengths and their direction—the redder the diamonds are, the stronger and more negative the associations are; the greener the diamonds are, the stronger and more positive the associations are; the grayer the diamonds are, the weaker the associations are. The stroke color of the diamonds (i.e., the “line color”) can be used to differentiate associations between behavioral beliefs with different determinants (attitude and intention in this case study). In this example, the diamonds with a red stroke show the association with attitude, and the diamonds with a blue stroke show the association with intention. The confidence intervals of the explained variance (*R*^2^) of attitude and intention based on all behavioral beliefs are depicted at the top of the figure. The items concerning behavioral beliefs can also be ranked based on association strengths with a specific determinant. In this example, the items are ranked based on their association with attitude. It would be overly simplified to use this ranking to select the most relevant sub-determinants (in this case behavioral beliefs). Instead, correlation coefficients, means, and confidence intervals of both need to be combined to select behavioral beliefs to be targeted in an intervention.

### Sub-Determinant Selection and Implications for Intervention Content

The procedure to select relevant sub-determinants and what this implies for intervention content is illustrated by using the visualizations regarding four items depicted in Figure 1. We selected four items that exhibit different univariate and bivariate patterns.

First, the item “For my health, using a high dose of ecstasy is… [much worse/much better; item 2 in Figure 1].” This belief has a strong positive association with attitude and intention. However, the relevance is relatively low, because the scores on the middle panel indicate that participants are already convinced that using a high dose of ecstasy is much worse for their health. If this belief is targeted in an intervention, then this would mean that the belief needs to be confirmed, unless it is possible to tailor the intervention message to only target the small subgroup of participants who are not convinced of the dose/risk relationship.

Second, the item “If I use a high dose of ecstasy, my trip is more… [mild/intense; item 15].” Although the scores on the middle panel indicate that participants are convinced that this makes their trip more intense, the relevance is relatively low, because this belief is not associated with attitude and very weakly with intention.

Third, the item “My experience with using a high dose of ecstasy is… [worse/better; item 1].” This belief has a strong positive association with attitude and intention, and the scores are on the middle of the scale. This combination makes it a highly relevant belief. In terms of intervening, this would imply that the belief that a high dose of ecstasy leads to a better experience needs to be negated. Other data from this study showed that common consequences of high doses of ecstasy, such as remembering less, hallucinating more, and being less sociable, were rated as very undesirable. This suggests that there may be enough leverage for a persuasive message that emphasizes the disadvantages of using a high dose.

Fourth, the item “Do you have as much, more, or less regret after using a high dose of ecstasy? [much less regret/much more regret; item 21].” This belief has a strong negative association with attitude and intention, and the scores are roughly normally distributed around the middle of the scale. This combination makes it a highly relevant belief. In terms of intervention, this would mean that feelings of regret need to be reinforced.

These four examples demonstrate the added value of combining several metrics (correlation coefficients, means, and confidence intervals of both) by means of visualizations. The next section is a practical guide on a freely available tool that can be used to obtain the visualizations needed to apply CIBER.

### A Practical Guide to Obtain Visualizations

We implemented this tool as a function in the open source package “userfriendlyscience” (39) for the open source statistical package R (40), which is often used in conjunction with the graphical user interface provided by the open source software RStudio (41). To use the function, the following commands can be used in an R analysis script or entered in the R console:

install.packages('userfriendlyscience');
require('userfriendlyscience');


The first of these commands downloads the package and installs it. This command only needs to be run once: the package will remain installed. The second command loads the package in the current session: this command has to be repeated in every session where the user wishes to use this package. After loading the package, the following command can be used to request the plot to apply CIBER:

CIBER(data = getData(),
    determinants = c('variable1',
            'variable2'
    targets = c('behavior', 'intention'));


In this simplest case, the first argument specifies the dataset to use. In this example, the function “getData” is used to load a dataset from, for example, an SPSS datafile. The second and third arguments are used to specify the variable names of the sub-determinants (which will appear in the rows of the plot) and the variable names of behavior and potentially other determinants. The associations of the sub-determinants with these latter variables will be shown in the panel to the right. Of course, more than two sub-determinants and targets can be specified by adding more variable names, delimited by commas and each enclosed in single (or double) quotes. Note that R is case sensitive, so the variable names have to match those in the datafile exactly.

The function has many optional arguments, the most relevant ones of which will briefly be listed here (use “?CIBER” to consult the function’s manual page). “subQuestions,” “leftAnchors,” and “rightAnchors” can be used to specify the items and anchors that were used to measure the sub-determinants. Setting “decreasing” to TRUE or FALSE orders the sub-determinants based on their means in descending or ascending order, respectively. If a target variable name is specified in the argument “orderBy,” then the sub-determinants are ordered by their association to that target variable instead. We refer to https://osf.io/qf3sq for the specific arguments used to create Figure 1 and the Supplementary Material for a general description of the arguments to be specified when using CIBER. All these optional arguments can be used to tailor the plots to the specific needs of a study aimed at selecting relevant sub-determinants.

## Discussion

The current paper demonstrates how to select the most relevant sub-determinants and how this can have an impact on choices made during intervention development (as demonstrated by the case study). We have described an analytical approach, denoted as CIBER, to look at associations between sub-determinants and (multiple) outcomes (e.g., behavior, but also determinants at lower levels of psychological aggregation). To facilitate the implementation of CIBER in future research, we have made an easy-to-use tool freely available, and we have described how to use it in practice. This tool provides the output needed to select relevant sub-determinants.

However, the utility of the output depends on the quality of the operationalizations (e.g., questions in the survey). Two aspects are crucial in ensuring high-quality operationalizations: (1) identifying all possible sub-determinants that need to be operationalized (e.g., included in the survey) and (2) making sure that they are adequately operationalized. With regard to the first aspect, we refer to the core processes (described in Section “Identifying Determinants at Different Levels of Psychological Aggregation”) that are critical to identify sub-determinants at all levels of aggregation (11). When identifying possible sub-determinants, it is warranted to be theoretically promiscuous and to remain critical toward all individual theories (9). After all, the aim is not to test a specific theory, but to identify all relevant aspects of the target population’s psychology where it concerns the behavior at hand (i.e., all possible sub-determinants). Hence, limiting oneself to only operationalize determinants in any one theory would be unwise. With regard to the second aspect, it is good to be aware that data about a determinant are only as good as its operationalization, and therefore, any theory should include instructions for operationalization of each determinant (42). For example, Witte et al. (43) provide a Risk Behavior Diagnosis Scale that contains skeleton items to be completed with the target behavior and health threat at hand, such as “[Recommended response] is effective in pre- venting [health threat].” Both aspects are essential to ensure utility of the output when applying CIBER.

Moreover, decisions regarding selection of sub-determinants cannot be solely based on the output of data analysis (regardless of which analytical approach is taken). The output should be seen as complementary to the expertise of a behavior change expert (e.g., health psychologist or health promoter). This expertise is needed to choose appropriate behavior change methods if a certain sub-determinant is selected and to translate these methods into practical applications (11). For example, providing stereotype-inconsistent information (i.e., positive examples from the stigmatized group) is a behavior change method aimed at reducing stigma. This method is only effective when there are many different examples, and these examples are not too discrepant from the original stereotype (11, 44). If providing stereotype-inconsistent information is operationalized in such a way that only few different examples are used, people might think that just one exception is presented. If the examples are too discrepant, people might deem the information to be irrelevant regarding their views on the stigmatized group. Expertise regarding parameters for use is crucial to adequately translate behavior change methods into practical applications.

Behavior change expertise is also needed to make judgments regarding changeability. Besides relevance, changeability is the other part of the equation when selecting sub-determinants during intervention development (1). For example, it is often assumed that knowledge about a certain behavior is relatively easy to change in comparison with self-efficacy toward that same behavior. Whenever possible, judgments regarding changeability should be based on evidence from the research literature (45). However, when data regarding changeability are scarce, such judgments have to rely on a theoretical or conceptual basis.

Finally, using CIBER might result in a large number of relevant (and changeable) sub-determinants. In practice, however, the available resources (e.g., time and money) are often limited. This can have an impact on the quantity and quality of intervention content that can be developed, but also delivered. The latter is especially relevant in case there are additional costs per participant (e.g., delivering an intervention in a face-to-face setting with a health professional). However, also when the additional costs per participants are low (e.g., when using an Internet-delivered intervention), then there are still limits in terms of the amount of intervention content that participants can be exposed to. Although intervention content can be delivered in multiple sessions over a longer period of time, this might lead to increased levels of dropout (46), which also limits exposure to intervention content. So, besides output of the proposed analytical approach and behavior change expertise, also practical constraints affect the ultimate selection of sub-determinants that are targeted in an intervention.

The proposed analytical approach can be applied at all levels of psychological aggregation. For example, as explained in Section “Identifying Determinants at Different Levels of Psychological Aggregation,” there are five levels of psychological aggregation in the RAA: behavior (level 1), intention (level 2), attitude (level 3), experiential attitude (level 4), and behavioral beliefs (level 5) (5, 9). Investigating associations between, for example, beliefs and intention/behavior might result in valuable insights, but also, for example, investigating associations between beliefs and attitude, or attitude and intention/behavior. The associations to look at depend on the exact question to be answered. For example, if one wants to know what the most relevant determinants are, then one needs to look at the associations between attitude, perceived norm, and perceived behavior control (level 3), and intention (level 2) or behavior (level 1). However, if one wants to know which beliefs to target in an intervention, then associations between beliefs (level 5), and intention (level 2) or behavior (level 1) are recommended. In the latter example, determinants such as attitude or perceived behavior control (level 3) are still important, because behavior change methods are linked to determinants at this level (11). For example, some methods are more appropriate to change attitude (e.g., arguments), while other are more appropriate to change perceived behavior control (e.g., guided practice). The exact content when applying these methods in an intervention, however, depends on the behavioral beliefs and control beliefs underlying, respectively, attitude and perceived behavioral control. Associations between beliefs (level 5) and determinants (level 3) shed more light on the latter. Independent of the levels of psychological aggregation one is interested in, CIBER can be applied to select the most relevant sub-determinants.

It needs to be stressed that conclusions regarding relevance of sub-determinants (e.g., when discussing the results of the case study presented in this paper) do not imply causality between sub-determinants and (multiple) outcomes. This is independent of the analytical approach, but is due to the cross-sectional nature of the data used in the study at hand. This is in line with the current literature that is dominated by cross-sectional determinant studies (47), although there are also experimental studies available [e.g., Ref. (48)]. Furthermore, we also do not want to imply that the associations between sub-determinants and outcomes are necessarily unidirectional in a theoretical sense. In fact, many theories assume a reciprocal relationship. For example, Bandura denotes this as reciprocal determinism in his Social Cognitive Theory (49). Weiner’s attributional model, as another example, indicates that unexpected or negative behavioral outcomes lead a person to search for causal ascriptions (e.g., specific beliefs) that can explain the outcomes (50). Longitudinal and experimental data are needed to test such assumptions.

In sum, CIBER is a useful approach to select the most relevant social-cognitive determinants, which can be applied across behavioral domains. Currently, however, CIBER is based on linear correlations between variables. That means that it cannot be applied yet to, for example, dichotomous variables. The underlying idea of the proposed analytical approach (i.e., using data visualization to combine several metrics regarding univariate distributions and associations), however, can also be applied to dichotomous variables. Therefore, we intend to continue to develop CIBER, to enable this in the future. Moreover, we are also aware of other developments, such as the use of network models that allow for modeling complex systems of observable items underlying psychological variables in general (51). These network models can also be applied to social-cognitive determinants, such as attitude (52). Within such network models, centrality measures (e.g., degree centrality and closeness centrality) might give complementary insights in the relevance of sub-determinants in relation to each other (53). The aim of this paper, however, was to demonstrate CIBER, not to compare it with other methods. Future research might focus on such comparisons and shed light on, for example, whether network models have added value on top of the proposed analytical approach. Using CIBER, however, is already a step forward from commonly used methods (e.g., regression analyses). To use CIBER, one needs to learn the rudiments of the statistical package R. However, the function is developed in such a way that substantive researchers can actually take this step and apply CIBER in future research and, therewith, optimize the development process of future behavior change interventions.

## Ethics Statement

The protocol for collecting the data that are used in the case study was approved by the ethical committee of the Dutch Open University (the form and the approval letter are available at https://osf.io/qf3sq). All participants gave online informed consent.

## Author Contributions

Study conception and design; drafting of manuscript: RC, G-JP, and JN; acquisition of data: G-JP and JN; analysis of data: G-JP; interpretation of data: RC and G-JP. All the authors agree on the final version of the manuscript.

## Conflict of Interest Statement

The authors declare that the research was conducted in the absence of any commercial or financial relationships that could be construed as a potential conflict of interest.

## Footnotes

^1^Please note that this does not imply that changes in the environment cannot have an impact on behaviour, only that this impact operates through psychological variables (either consciously or unconsciously).

^2^Peters G-JY, Noijen J. *Harder, Better, Faster, Stronger: Informing Behavior Change Interventions to Discourage Using High Doses of Ecstasy* (forthcoming).
