# Supplementary material for: Using Confidence Interval-Based Estimation of Relevance to Select Social-Cognitive Determinants for Behavior Change Interventions
Source: Front Public Health. 2017 Jul 13;5:165. doi: 10.3389/fpubh.2017.00165 (PMC5508122; doi:10.3389/fpubh.2017.00165)
Supplement: Supplementary file 1 [file Table_1.docx]

Supplementary Material

Using Confidence Interval-Based Estimation of Relevance to select social-cognitive determinants for behaviour change interventions

Rik Crutzen*, Gjalt-Jorn Ygram Peters, Judith Noijen

*** Correspondence:** Corresponding Author: [rik.crutzen@maastrichtuniversity.nl](mailto:rik.crutzen@maastrichtuniversity.nl)

**Specifications of arguments when using CIBER**

This Supplementary Material describes the specifications when using CIBER. Note that more supplementary materials are available at the Open Science Framework at <https://osf.io/qf3sq>. In this brief overview of specifications presented below, a distinction has been made between required arguments and additional arguments.

| **Required arguments** | |
| --- | --- |
| data | The dataframe containing the variables. Use getData() to load a dataset from an SPSS datafile. |
| determinants | The ’determinants’: the predictors (or ’covariates’) of the target variables(s) (or ’criteria’). |
| targets | The ’targets’ or ’criteria’ variables: the variables predicted by the determinants. |
| **Additional arguments** | |
| conf.level | The confidence levels for the confidence intervals: has to be a named list with two elements: means and associations, specifying the desired confidence lev- els for the means and associations, respectively. The confidence level for the associations is also used for the intervals for the proportions of explained variance. If this argument is not specified, then the following defaults are used:   - Default confidence interval for means: 99.99% - Default confidence interval for associations: 95% |
| subQuestions | The questions used to measure each determinant. These are used as labels on the left of the figure. |
| leftAnchors/ rightAnchors | The anchors to display on the left/right side of the left hand panel. These can be used to show the anchors that were used for the respective scales. |
| orderBy | This can be used to specify whether to sort the determinants. Set to NULL to not sort at all (this is the default); specify the name one of the targets to sort by the point estimates of the associations with that target variable. |
| decreasing | This can be used to specify whether to sort the determinants. Specify NULL to not sort at all (this is the default), TRUE to sort in descending order, and FALSE to sort in ascending order. |

This Supplementary Material is based on the manual of the R package ‘userfriendlyscience’. This extensive manual also describes arguments to alter aesthetics (e.g., use of colours, font size) when using CIBER as well as other functions (besides CIBER) that are available in the R package. The complete manual for that package is available at:

<http://cran.r-project.org/web/packages/userfriendlyscience/userfriendlyscience.pdf>

Several websites present pages where the manual pages for specific functions are extracted and presented, such as these two:

<https://rdrr.io/cran/userfriendlyscience/man/CIBER.html>
<https://www.rdocumentation.org/packages/userfriendlyscience/versions/0.6-1/topics/CIBER>
